# Noninvasive Multimodal Imaging to Predict Recovery of Locomotion after Extended Limb Ischemia

**DOI:** 10.1371/journal.pone.0137430

**Published:** 2015-09-14

**Authors:** Jason S. Radowsky, Joseph D. Caruso, Rajiv Luthra, Matthew J. Bradley, Eric A. Elster, Jonathan A. Forsberg, Nicole J. Crane

**Affiliations:** 1 Department of Surgery, Uniformed Services University of Health Sciences, Bethesda, MD, United States of America; 2 General Surgery, Walter Reed National Military Medical Center, Bethesda, MD, United States of America; 3 Regenerative Medicine Department, Naval Medical Research Center, Silver Spring, MD, United States of America; 4 Orthopaedics and Rehabilitation, Walter Reed National Military Medical Center, Bethesda, MD, United States of America; 5 Henry M. Jackson Foundation for the Advancement of Military Medicine, Bethesda, MD, United States of America; University of New Mexico HSC, UNITED STATES

## Abstract

Acute limb ischemia is a common cause of morbidity and mortality following trauma both in civilian centers and in combat related injuries. Rapid determination of tissue viability and surgical restoration of blood flow are desirable, but not always possible. We sought to characterize the response to increasing periods of hind limb ischemia in a porcine model such that we could define a period of critical ischemia (the point after which irreversible neuromuscular injury occurs), evaluate non-invasive methods for characterizing that ischemia, and establish a model by which we could predict whether or not the animal’s locomotion would return to baselines levels post-operatively. Ischemia was induced by either application of a pneumatic tourniquet or vessel occlusion (performed by clamping the proximal iliac artery and vein at the level of the inguinal ligament). The limb was monitored for the duration of the procedure with both 3-charge coupled device (3CCD) and infrared (IR) imaging for tissue oxygenation and perfusion, respectively. The experimental arms of this model are effective at inducing histologically evident muscle injury with some evidence of expected secondary organ damage, particularly in animals with longer ischemia times. Noninvasive imaging data shows excellent correlation with post-operative functional outcomes, validating its use as a non-invasive means of viability assessment, and directly monitors post-occlusive reactive hyperemia. A classification model, based on partial-least squares discriminant analysis (PLSDA) of imaging variables only, successfully classified animals as “returned to normal locomotion” or “did not return to normal locomotion” with 87.5% sensitivity and 66.7% specificity after cross-validation. PLSDA models generated from non-imaging data were not as accurate (AUC of 0.53) compared the PLSDA model generated from only imaging data (AUC of 0.76). With some modification, this limb ischemia model could also serve as a means on which to test therapies designed to prolong the time before critical ischemia.

## Introduction

Tourniquets are regularly applied to lower limbs to limit blood loss in the operating room from surgical procedures as well as on the battlefield following blast injuries. The local physiologic and pathologic effects of vascular injury to a limb have been well studied [1, 2] and there is substantial evidence that longer ischemia times correlate with reduced functional outcomes. Furthermore, the deleterious effects of ischemia and reperfusion are not limited to the affected limb alone, but have systemic sequelae from released myoglobin and inflammatory cytokines, to including multi-organ dysfunction and even death.[3]

Commonly, the time to release of tourniquets and vascular clamps, with the subsequent restoration of blood flow to the limb, is the only identified information about limb reperfusion. Little is known, however, about the oxygenation of the distal tissue following release of vessel occlusion. Assessment is commonly subjective during operative evaluation and experienced surgeons apply the “four Cs” in an effort to determine whether tissue is adequately perfused and likely to recover. Recently, Forsberg and coworkers demonstrated that, for combat wounds, the “four Cs” are not adequate for portending wound outcome.[4] However, imaging modalities, which can ascertain perfusion and oxygenation, would lend insight into the viability of tissue and could help direct further vascular interventions or surgical debridement including the role of limb salvage in borderline cases.

Recently, imaging techniques have been employed in the operating room to ascertain adequacy of perfusion, including indocyanine green angiography, laser Doppler imaging, and laser speckle contrast imaging. The imaging technologies we present here, in general, are low in cost, non-invasive, rapid, and reliable. 3CCD (charge coupled device) imaging, a relatively ubiquitous imaging technology widely used in operating room suites, provides chemically specific information (oxygenated/deoxygenated hemoglobin), a large field of view, and *in vivo* detection simultaneously. Long wave infrared (IR) imaging in the range of 7.5–13.5μm provides data for overall tissue perfusion and has been proven experimentally to assess tissue function and metabolism.[5] In addition to their low cost in comparison to other imaging technologies, both 3CCD imaging and long wave IR imaging do not rely on extrinsic enhancement with injectable dyes or contrast agents.

By combining available and inexpensive technologies, such as 3CCD and long wave IR imaging cameras, we hypothesized that information concerning the perfusion, metabolism, and oxygenation of tissue can be ascertained in real-time. The following study is designed to discover the characteristics of limb tissue following variable ischemic periods as evaluated with 3CCD and IR imaging. Ischemia times included 3.5 and 4.7 hours, yielding moderate to severe ischemia/reperfusion injury (IRI). In a previous study by Burkhardt et al, ischemia times of 1, 3, and 6 hours were investigated; the investigators postulated that critical ischemia would be achieved with 4.7 hours of ischemia.[6] If successful, this technology could be used in a variety of settings, including the battlefield, to help identify the point of critical ischemia, which, in turn would guide surgical decision-making.

## Materials and Methods

### Ethics Statement

The study protocol was approved by the Institutional Animal Care and Use Committees at the Uniformed Services University of Health Sciences and the Naval Medical Research Center (protocols SUR-11-816 and 11-OUMD-13, respectively) in compliance with all Federal regulations governing the protection of animal subjects.

### In vivo studies

In this protocol, 26 adolescent female swine (*Sus scrofa*) ranging from 45–55 pounds were randomized to sham (n = 5), 3.5 hour tourniquet (n = 5), 3.5 hour occlusion (n = 5), 4.7 hour tourniquet (n = 5), and 4.7 hour occlusion (n = 6) experiment arms (Table 1). Each animals was housed in a single run and provided regular access to food and water, as well as environmental enrichment.

**Table 1:** Summary of experimental design.

Group	Ischemia Time (hours)	Ischemia Mode	N
1	0	none	5
2	3.5	occlusion	5
3	3.5	tourniquet	5
4	4.7	occlusion	6
5	4.7	tourniquet	5
		Animals expired	2
		Equipment Malfunction	2
		**Total Animals**	22

In all cases, the animals were anesthetized using intramuscular Telazol (4-6mg/kg IM, Fort Dodge Animal Health, Overland, KS, USA) and Dexdomitor (0.05 mg/m^2^ IM, Zoetis, Madison, NJ, USA) for initial sedation and then maintained with inhaled isofluorane (1.5%-3% with approximately 30% FiO_2_). A Foley catheter was inserted to collect urine and a tunneled central venous catheter (CVC) was placed in the right external jugular vein to allow for regular collection of blood samples. To reduce the risk of thrombosis formation during ischemia, unfractionated heparin (100U/kg) was administered prior to limb occlusion. Additonally, all animals received intravenous antibiotics (5 mg/kg, Baytril 100, Bayer Healthcare LLC, Shawnee Mission, KS) at the beginning of the surgical procedure and 3 days post-operatively. All animals in the sham and tourniquet groups had an incision in the right inguinal crease with exposure, but not clamping of the femoral vasculature to control for any amount of inflammation observed from the incision itself. The incision was made on the right hind limb because tourniquets were placed on the left hind limb (in the appropriate groups). For animals in the tourniquet experimental groups (later termed tourniquet animals), a pneumatic tourniquet (PediFit, Delfi Medical Innovations, Vancouver BC, Canada) was placed as proximally as possible on the left lower extremity and connected to a portable inflation system (PTSii, Delfi Medical Innovations, Vancouver BC, Canada) and inflated to 250 mm Hg. For animals in the 3.5-hour occlusion group (later termed 3.5 hour occlusion animals), occlusion was achieved by direct clamping of the proximal femoral artery and vein at the level of the inguinal ligament. Both the artery and vein were occluded to approximate the complete occlusion of efferent and afferent flow induced by the tourniquet. With the assistance of the thermal camera we adjusted the vascular clamp proximally, if needed, until collateral flow was absent; swine, however, have an extremely redundant vascular supply in their hind limbs and complete ischemia (100%) of the limb was often not achieved as perfusion was sometimes observed in small proximal and lateral vessels of the limb. Occlusion in the 4.7-hour group was accomplished by clamping both the internal and external iliac vasculature at their branch-point from the aorta via a left lower quadrant incision and extraperitoneal dissection, required to ensure complete ischemia of the limb (these animals are termed 4.7 hour occlusion animals). Animals were administered Fentanyl (75–100 μg/kg/hr transdermal patches) and buprenorphine (0.03 mg/kg intramuscular injections) operatively and post-operatively for analgesia. Animals were euthanized prior to the study endpoint if they met criteria for deterioration of clinical condition, including a significantly decreased or elevated heart rate, respiration rate, body temperature, or arterial pressure; the inability to ambulate for more than 24 hours; and/or the inability to eat or drink water.

### Clinical and Laboratory Data Acquisition

Heart rate, body temperature, respiratory rate, end tidal CO_2_ and oxygen saturation were monitored throughout the procedure. Blood and urine samples were collected at every hour and after reperfusion the day of the procedure, as well as on Day 1 (D1), Day 3 (D3) and Day 7 (D7). Whole blood was used for a complete blood count (CBC) and blood serum was used for chemistry analysis as well as serum cytokine analysis. The blood chemistry panel included a basic metabolic panel, calcium, phosphorus, cholesterol, triglycerides, total protein, albumin, AST, ALT, LDH, creatine kinase, ALKP, GGT, and total bilirubin. Blood urea nitrogen was first converted to serum urea content (mmol/L) prior to normalizing analytes of interest to serum urea content (U/mmol). Urine was analyzed for urea, creatinine, pH, specific gravity, and presence of blood or protein.

Serum cytokines were analyzed using multiplex ELISA kits (Aushon Biosystems, Billerica, MA). Cytokines measured included IFNα, IFNγ, IL-1β, IL-2, IL-4, IL-6, IL-8, IL-10, and TNFα.

Core needle biopsies (14g) were taken from the injured leg at baseline, 30 minutes post-reperfusion, D1, D3 and D7; each was stored in 10% neutral buffered formalin prior to histologic preparation. Clinical observations were recorded twice daily for seven days. Each animal was evaluated by general clinical appearance, food and water consumption, and behavior. The resulting extremity injuries were scored during clinical observations with a modified Tarlov scale[7] to determine how ischemia affected locomotion (0: complete paralysis, 1: minimal movement, 2: stands with assistance, 3: stands alone, 4: weak walk, 5: normal gait). A Tarlov score of 5 indicated normal mobility while a Tarlov score of 2 was manifested by significantly impaired mobility.

After collection of D7 blood and urine samples, animals were euthanized by intravenous injection of sodium pentabarbital (100 mg/kg, Beuthanasia, Merck Animal Health, Madison, NJ). Muscle samples were taken from predetermined proximal, mid, and distal sections of the bilateral hind-limbs, both from the medial and lateral aspect. Pieces of the porcine liver, kidney, proximal stomach and esophagus and lung were also collected and submitted for pathologic analysis. Samples were graded from 0–5 based on the presence of edema, non-suppurative inflammation, degeneration, necrosis, and regeneration in the submitted tissue by veterinary pathologists attached to Naval Medical Research Center; pathology scores are the sum of each category (i.e. 1-edema + 1-inflammation + 1-degenration + 1-necrosis + 1-regeneration = 5). A pathology score of 25 indicates the highest level of injury in tissue; a pathology score of 0 indicates no injury in tissue.

The animals were observed twice a day for seven days. Their behavior was rated on general clinical appearance, food and water consumption, and behavior. The Tarlov scale was also used to determine how ischemia affected locomotion. Additionally, a drop foot score was used to monitor neuropathy in the affected limb.

At study endpoint (D7) a necropsy was performed to harvest tissue (kidney, liver and skeletal muscle) and placed in 10% neutral buffered formalin. Other organs were harvested if evidence of damage was observed, indicated by gross pathology. The tissues were then submitted for paraffin embedding, sectioning, mounting of sections and staining of slides with hematoxylin and eosin (H&E) to monitor morphological change in tissue.

### Non-invasive Imaging

**Fig 1:**
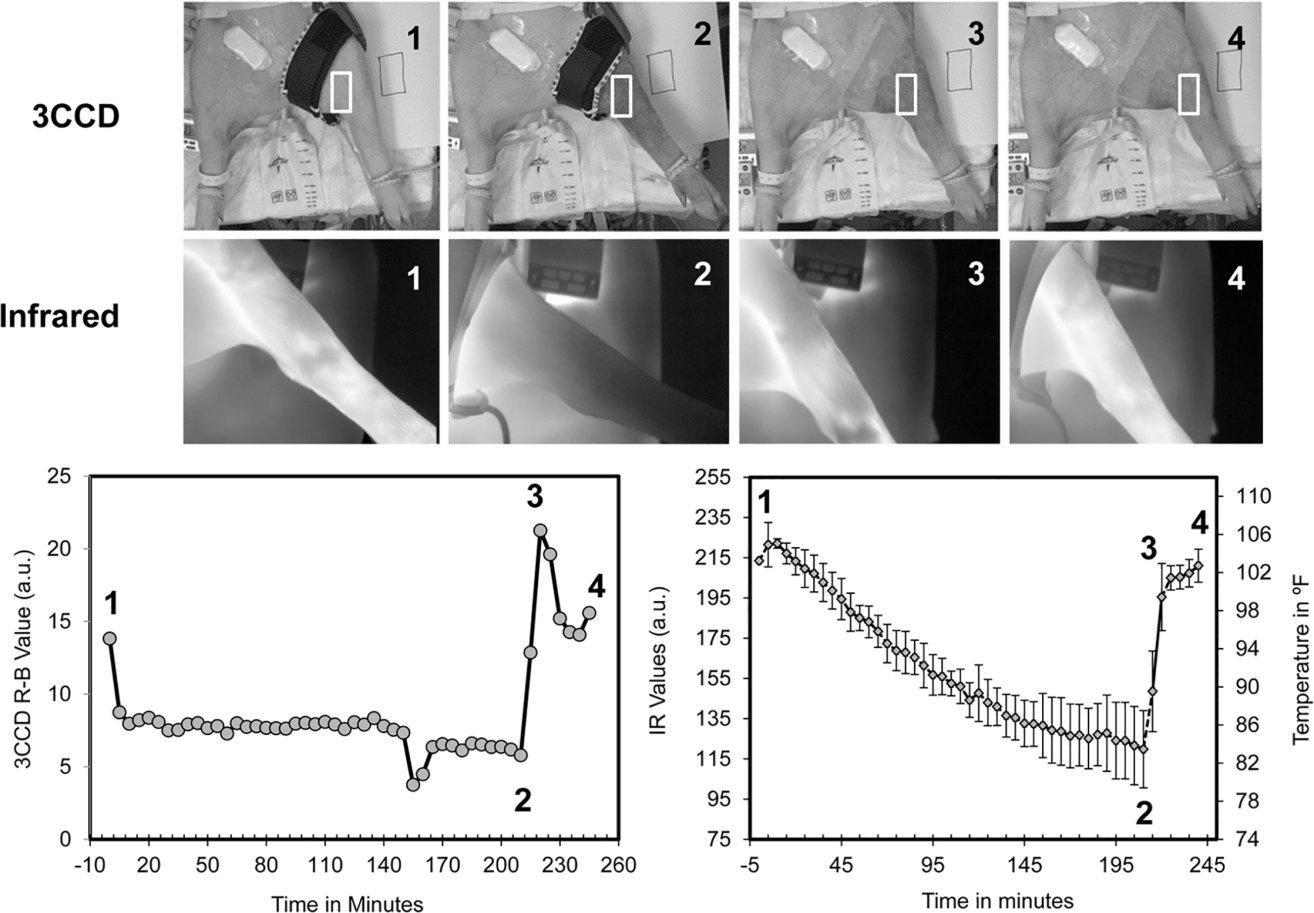
3CCD and infrared (IR) imaging. Top) Representative grayscale 3CCD images of a hind limb, and Middle) representative grayscale infrared images of a hind limb at (1) baseline, (2) maximum ischemia, (3) 10 minutes post-reperfusion, and (4) 30 minutes post-reperfusion. Bottom left) Profile of R-B values, derived from 3CCD imaging, over the course of 3.5 hours of ischemia and 30 minutes of reperfusion. Bottom right) Profile of IR values and corresponding mean leg temperature over the course of 3.5 hours of ischemia and 30 minutes of reperfusion. The time points mentioned previously (1–4) are noted.

Throughout the procedure, images and spectral data were collected using both 3CCD and infrared (IR) modalities. For this study, a commercially available 3CCD camcorder (HDC-HS9) was used to document the entirety of the procedure. The camera was white balanced at the beginning of the procedure, and frames of interest were extracted from the video files. Additionally, IR images were collected using a FLIR Tau640 (Santa Barbara, CA, USA) camera and transferred to a PC using a frame grabber card (Frame Link Express, VCE-CLEX01, Imprex, Boca Raton, FL). Images were collected at five-minute intervals during the entire procedure. An in-house developed tool allowed for tracking of a region of interest (ROI) over the duration of the procedure. Representative images are displayed in Fig 1 (top panels) for 4 time points (t = 0, t = 210, t = 220, and t = 240 minutes, respectively). ROIs are indicated by a white box. The normalized change in 3CCD values was plotted as a function of time (Fig 1, bottom left panel). A similar process was used to track changes in IR values (Fig 1, bottom right panel). Equipment malfunctioned during two of the cases and 3CCD imaging data was not collected.

### Data Analysis

All image data acquired was processed using custom Matlab (Mathworks, Nattick, MA, USA) programs. Images were registered and subsequently processed, tracking a selected ROI in both the IR and 3CCD image frames. In the IR frames, a mean intensity value for the ROI was used to assess limb perfusion.[8] Values were converted to temperature using a calibration for mean IR values and temperature. For the 3CCD frames, mean red channel minus blue channel (R-B) values correlated to tissue oxygenation.[9–13] The percent difference of R-B values from the baseline R-B value was calculated for each time point.

The slope of reperfusion was determined by the difference between the 3CCD and IR maximum ischemia values and their corresponding maximum reperfusion values divided by the duration of reperfusion. The slope of post-occlusive reactive hyperemia (PORH) was calculated from the difference between the 3CCD and IR maximum reperfusion values and their corresponding 5 or 10 minute post-reperfusion values divided by the duration of the post-reperfusion period.

### Statistical Analysis

One-way analysis of variance (ANOVA) was used to compare differences across groups for normally distributed continuous data, with the post-hoc Bonferroni correction employed to compare differences between individual groups. For comparisons of tourniquet and occlusion animals or a comparison of fully recovered and not fully recovered animals, a Kruskal-Wallis ANOVA was used. P-values less than 0.05 were considered statistically significant and are indicated by an asterisk (*); p-values < 0.01 were considered very statistically significant and are indicated by a double asterisk (**); p-values < 0.001 were considered the most statistically significant and are indicated by a triple asterisk (***). Bivariate correlations were assessed using Spearmans correlation coefficient. Correlation coefficients greater than 0.7 (positive or negative) were considered important. All univariate statistics were analyzed using IBM SPSS v.22 (IBM Corp, Armonk, NY).

Partial-least squares discriminant analysis (PLSDA) was performed in PLS_Toolbox (Eigenvector Research Inc., Wenatchee, WA). Briefly, PLSDA is a classification algorithm that places samples into two groups and then places a line between the two groups to discriminate the two groups of samples. In this study, we use multiple variables to create this discrimination and the line becomes a hyperplane separating the two groups of samples in a multidimensional space. Here, the two groups are 1) returned to normal locomotion and 2) did not return to normal locomotion. Variables were mean-centered and one or two principal components. Cross-validation was performed with random subsets of samples, including 4 data splits and 10 iterations. Samples with excessive missing data points were excluded from the model.

## Results

Two animals expired before the one-week survival period (one sham–acute lung injury; one 4.7 hour occlusion–did not regain consciousness) and one animal was euthanized prematurely (3.5 hour tournuiquet) on post-operative day 5; data points for intraoperative and post-reperfusion were retained in the data set, if collected.

### Blood Chemistries Exhibit End Organ Stress

**Fig 2:**
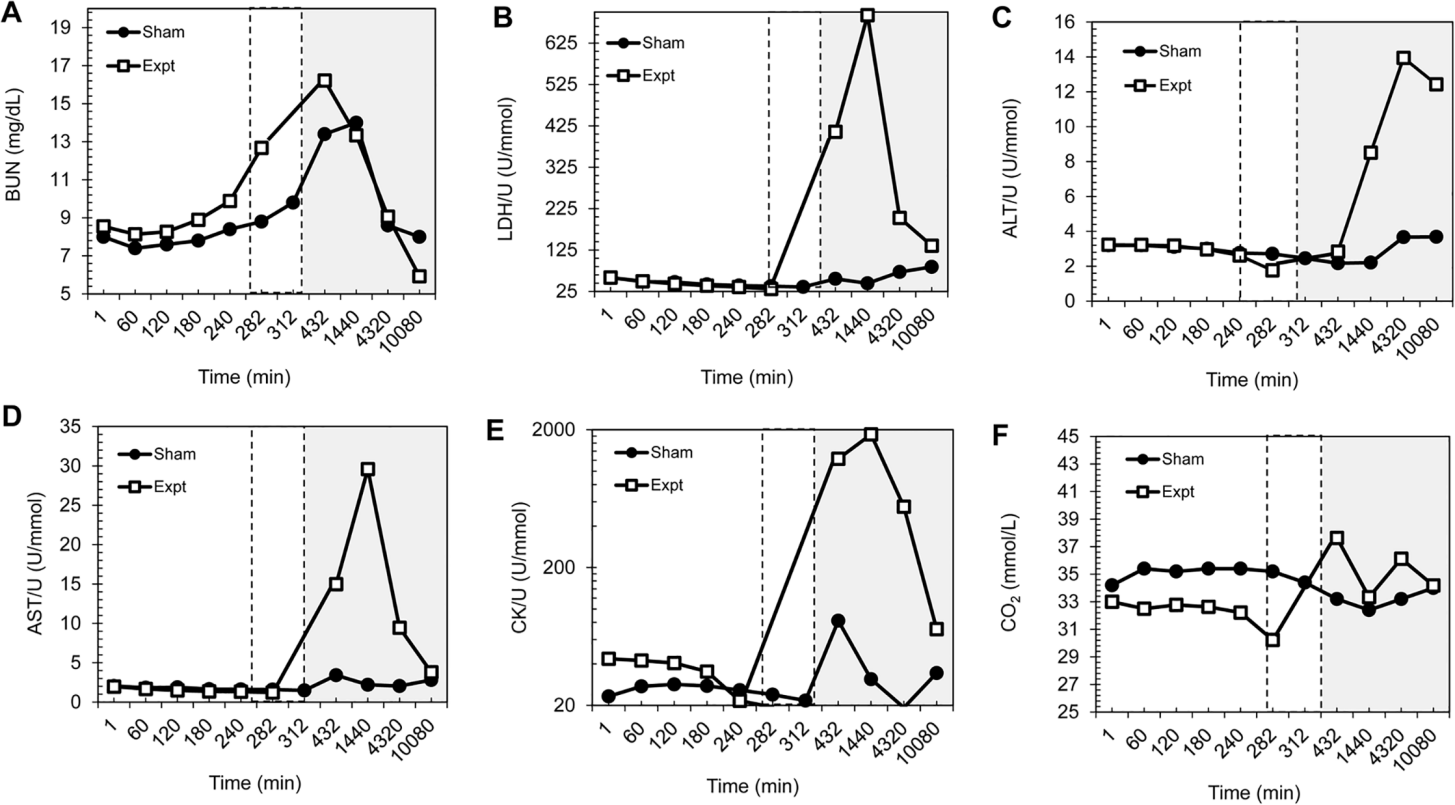
Comparison of blood chemistries in sham and experimental ischemia animals. Comparison of blood chemistries (A-E) for sham animals (black circles) and experimental, or ischemic, animals (white squares). The dashed window indicates the region of maximum ischemia and reperfusion, while the gray window highlights post-operative time points. All time points are plotted as minutes; 1440 min = D1, 4320 min = D3, and 10,080 min = D7. All blood chemistries have been normalized to blood urea with the exception of CO_2_, which prevents erroneous data trends due to dehydration. A) BUN = blood urea nitrogen; B) LDH = lactate dehydrogenase; U = urea; C) ALT = alanine aminotransferase; D) AST = aspartate aminotransferase; E) CK = creatinine kinase; F) CO_2_ = carbon dioxide = bicarbonate.

Blood samples were analyzed with the standard Chem20 panel. Blood urea nitrogen (BUN) served as a marker of renal injury. In Fig 2A, BUN levels begin to increase towards the end of ischemia and continue to increase post-operatively, peaking at 3 hours post-reperfusion, however, these values are not appreciably different from BUN levels in sham animals. Lactate dehydrogenase (LDH) in this study served as a marker of general tissue damage and injury. LDH increased at 3 hours post-reperfusion and peaked on D1 (Fig 2B). Aminotransferases, specifically alanine aminotransferase (ALT) and aspartate aminotransferase (AST), were used to monitor liver function and were elevated post-operatively in tourniquet and occlusion animals (Fig 2C and 2D). ALT levels increased at D1 for all tourniquet and occlusion animals, reached its apex at D3, but continued to stay elevated to D7 for tourniquet animals only. AST levels began to increase at 3 hours port-reperfusion, peak at D1, and returned to baseline levels by D7. Creatine kinase (CK) is released into the bloodstream when damage is incurred in muscle and thus is used as a serum marker of skeletal muscle injury. CK levels have a similar profile to AST levels; CK levels increase by 3 hours post-reperfusion, peak at D1, and return to near baseline levels by D7 (Fig 2E). Finally, bicarbonate levels (measured as CO_2_) in the blood can be a determinant of oxidative damage after IRI. In occlusion and tourniquet animals, bicarbonate levels decreased at 30 minutes post-reperfusion and continued to decrease within the first 24 hours, but return to baseline levels by 24 hours post-reperfusion (Fig 2F).

As expected, animals in all experimental groups demonstrated higher levels of AST (p<0.05), ALT (p<0.001), LDH (p<0.05), and CK (p<0.03) at post-operative D1 when compared to sham animals. Animals in the 4.7 hour tourniquet group demonstrated higher levels of AST (p<0.03) and CK (p<0.03) on D3 compared with sham animals. Potassium was elevated in all experimental animals compared to sham animals 1 week post-operatively (p<0.01). Hematologic and renal parameters were not significantly different across groups. BUN levels at maximum ischemia were significantly higher in animals that did not fully recover locomotion when compared to animals that did fully recover locomotion (p = 0.05). All other blood chemistry levels showed no significant difference between animals without fully recovered locomotion and animals with fully recovered locomotion (Table 2).

**Table 2:** Outcome based comparison of various metrics. Values are means +/- the standard deviation for animals that 1) recovered full locomotion, and 2) did not recover full locomotion. Differences approaching statistical significance (p-value ≈ 0.1) are indicated by (†). Statistically significant differences (p-value<0.05) are indicated by an asterisk (*).

Metric	Mean +/- s Recovered Fully	Mean +/- s Did Not Recover Fully	p- value
Tarlov D1	4.42 ± 1.16	3.50 ± 0.76	0.07†
Tarlov D2	4.67 ± 0.49	3.81 ± 0.37	0.0006*
Tarlov D3	4.75 ± 0.45	3.88 ± 0.35	0.0002*
Tarlov D7	5.00 ± 0.00	3.94 ± 0.82	0.0004*
Left Kidney Pathology	0.08 ± 0.29	0.50 ± 0.53	0.04*
Right Kidney Pathology	0.08 ± 0.29	0.50 ± 0.53	0.04*
Liver Pathology	0.17 ± 0.39	0.13 ± 0.35	0.81
Drop Foot	0.08 ± 0.29	1.13 ± 0.99	0.003*
ALT Max Isc	70.6 ± 13.8	63.3 ± 11.1	0.22
AST Max Isc	37.3 ± 11.4	35.1 ± 18.0	0.75
Creatinine Max Isc	1.13 ± 0.17	1.18 ± 0.28	0.63
BUN Max Isc	9.00 ± 2.66	11.3 ± 1.83	0.05*
K+ Max Isc	5.24 ± 0.85	5.68 ± 0.93	0.30
LDH Max Ischemia	1072 ± 367	984 ± 217	0.54
CK Max Isc	1010 ± 1765	631 ± 309	0.56
ALT Rpf	70.3 ± 11.8	63.6 ± 10.9	0.22
AST Rpf	42.6 ± 14.8	43.1 ± 13.1	0.93
Creatinine Rpf	1.16 ±0.18	1.10 ± 0.33	0.61
BUN Rpf	9.75 ± 2.86	11.00 ± 3.02	0.36
K+ Rpf	5.44 ± 0.78	6.01 ± 0.89	0.15
LDH Rpf	1126 ± 388	1053 ± 288	0.65
CK Rpf	732 ± 463	1077 ± 545	0.15
O2 Sat Max Isc	96.3 ± 2.6	98.0 ± 2.0	0.13
O2 Sat Rpf 5min	96.2 ± 2.2	97.6 ± 1.5	0.12
Temp Max Isc	99.5 ± 5.9	101.9 ± 1.0	0.27
Temp Rpf 5 min	99.6 ± 5.9	101.9 ± 1.0	0.28
Pulse Max Isc	101.8 ± 14.5	99.1 ± 5.7	0.62
Pulse Rpf	101.9 ± 14.8	99.0 ± 5.9	0.60
sO2 Max Isc	77.1 ± 4.5	81.4 ± 3.5	0.06†
sO2 Rpf 30 min	78.3 ± 4.2	78.1 ± 7.0	0.95
IR Slope Rpf	3.71 ± 8.98	10.60 ± 13.64	0.25
IR Whole Leg Rpf5	155.6 ± 48.1	105.8 ± 32.3	0.04*
IR Whole Leg Rpf10	187.3 ± 24.3	152.0 ± 36.3	0.05*
IR Whole Leg Rpf30	203.4 ± 13.5	191.6 ± 16.9	0.17
IR PORH Slope Rpf5	4.74 ± 3.91	2.55 ± 6.91	0.46
IR PORH Slope Rpf10	3.82 ± 4.79	5.82 ± 4.28	0.44
IR PORH Slope Rpf30	2.09 ± 1.25	4.07 ± 1.14	0.01*
3CCD Slope Rpf	0.44 ± 0.73	0.90 ± 0.62	0.18
3CCD Whole Leg Rpf10	29.3 ± 10.3	30.8 ± 8.4	0.75
3CCD Whole Leg Rpf30	26.7 ± 11.6	27.6 ± 6.4	0.85
3CCD PORH Slope Rpf5	-0.11 ± 0.36	-0.38 ± 0.28	0.10†
3CCD PORH Slope Rpf10	-0.28 ± 0.22	-0.36 ± 0.21	0.48

### Serum Cytokines Reveal Little Regarding Return to Full Locomotion

Serum cytokine levels were measured in all sham and experimental animals and included a panel of nine cytokines and chemokines associated with inflammation (pro-inflammatory and anti-inflammatory). Cytokines levels were highly variable within experimental groups, making an accurate comparison difficult. Few statistically significant differences were observed between experimental groups (data not shown). IL-13 was significantly elevated in tourniquet animals compared to occlusion and sham animals (p<0.02).

### Evidence of IRI and Attenuated Locomotion

**Fig 3:**
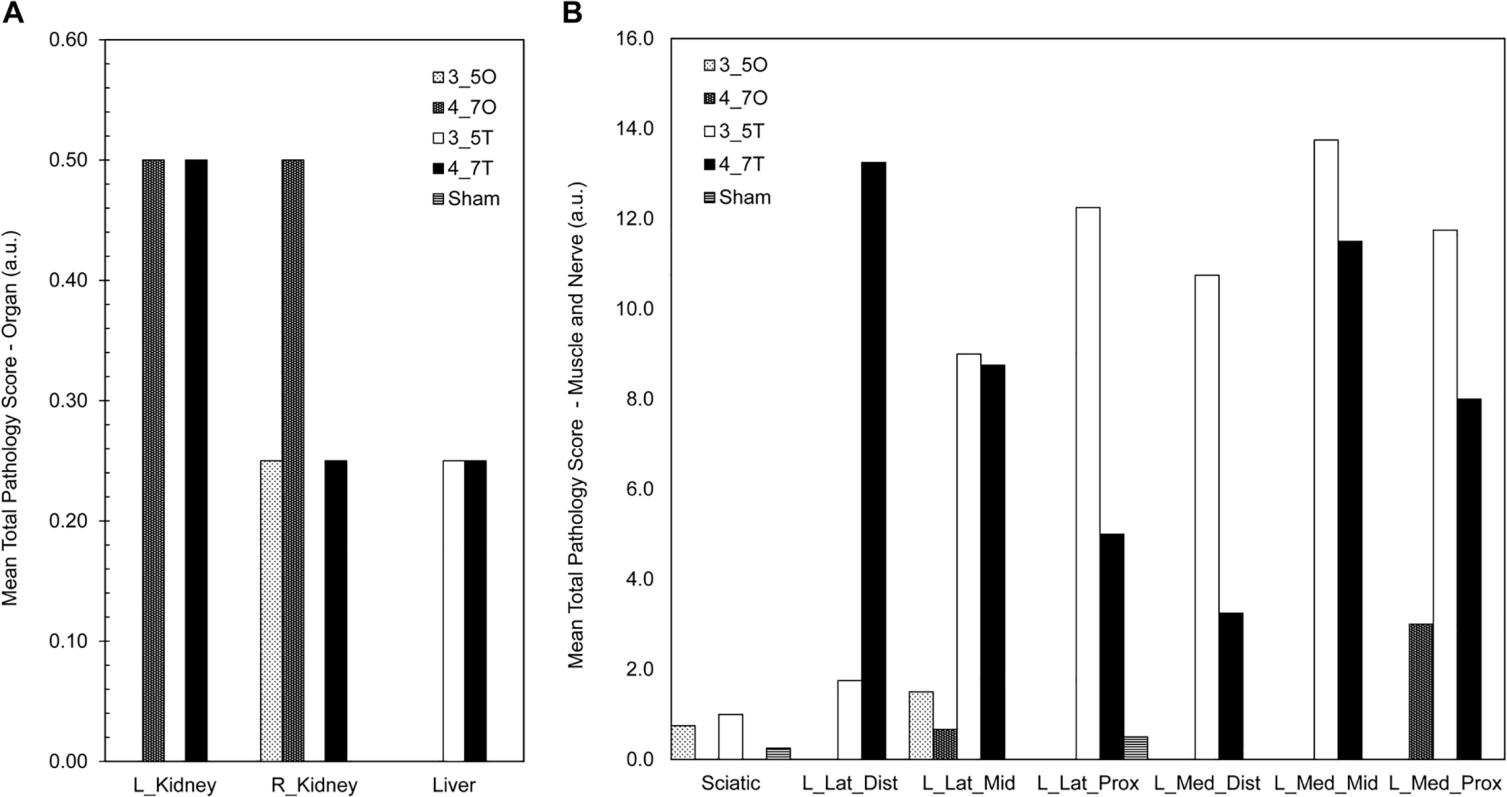
Mean pathology scores for all experimental groups. Mean total pathology scores for all experimental groups: A) organs (kidneys and liver), and B) sciatic nerve and skeletal muscle. 3_5O = 3.5 hour occlusion; 3_5T = 3.5 hour tourniquet; 4_7O = 4.7 hour occlusion; 4_7T = 4.7 hour tourniquet.

Mean pathology scores for the left and right kidneys and liver are presented in Fig 3A. Some end organ damage was observed in both the left and right kidneys of the 4.7 hour occlusion animals and the kidneys and liver of the 4.7 hour tourniquet animals. A small degree of damage was also observed in the liver of the 3.5 hour tourniquet animals, though not consistently. When comparing the mean pathology scores for muscle and sciatic nerve, we saw increased pathology scores for all tourniquet animals compared to occlusion animals–Fig 3B. The patterns of damage to the skeletal muscle were not, however, consistent across both cohorts of occlusion and tourniquet animals.

**Fig 4:**
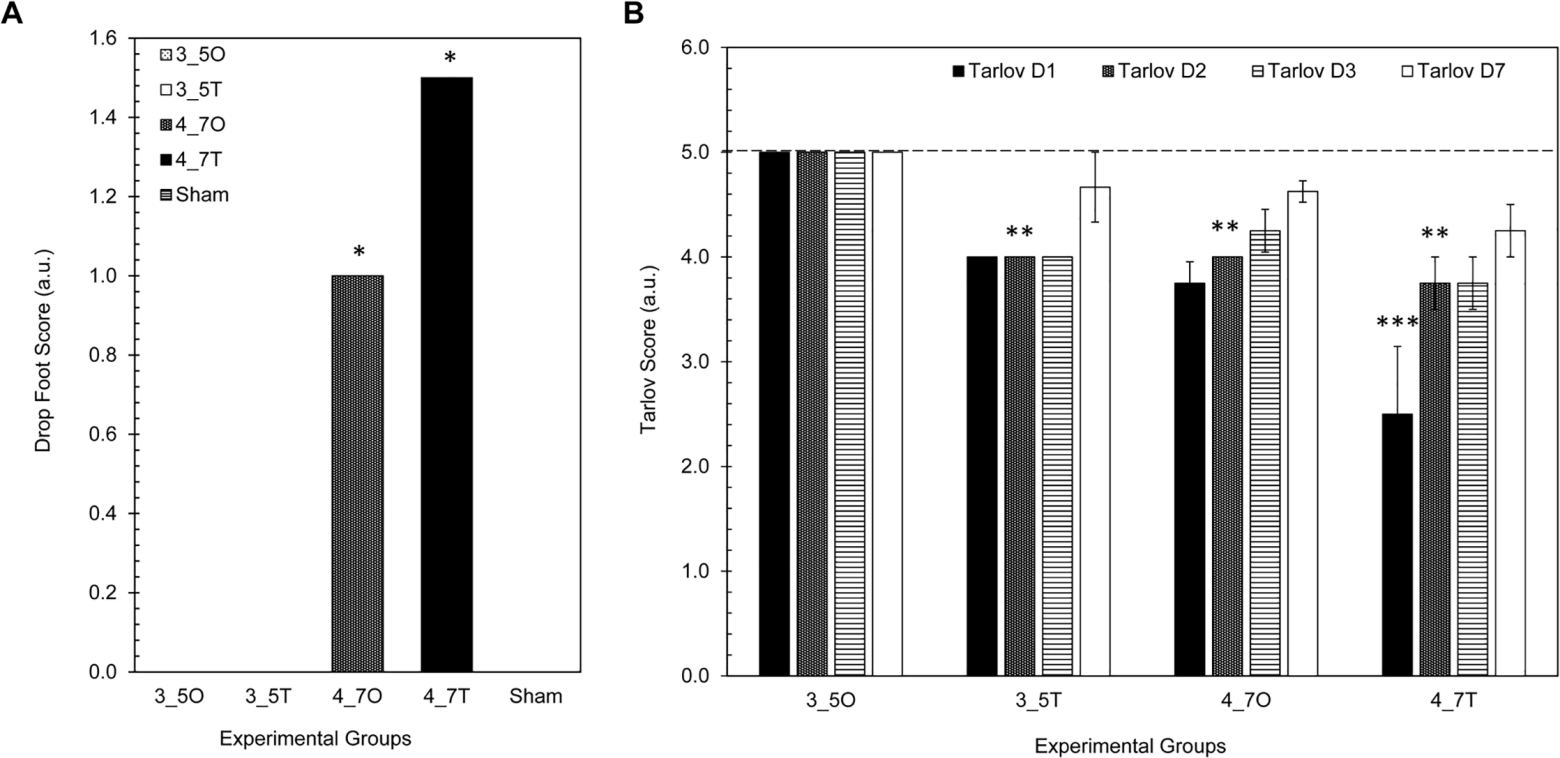
Functional outcomes in sham and experimental ischemia groups. A) Mean drop foot scores for all experimental groups. A drop foot score of 0 meant that no neuropathy was observed post-operatively. A drop foot score of 1 was consistent with neuropathy in the affected limb for at least one day post-operatively. A drop foot score of 2 was consistent with neuropathy in the affected limb for at least three days post-operatively. A drop foot score of 3 was indicative of neuropathy in the affected limb beyond 5 days post-operatively. B) Mean Tarlov scores for all experimental groups. The dashed line at 5.0 is reflective of normal locomotion. The asterisks indicate statistically significant differences when compared to sham and 3.5 hour occlusion animals—** = p-value < 0.01, *** = p-value < 0.001.

All animals in the 4.7 hour occlusion and 4.7 hour tourniquet groups had an increased drop foot score when compared to sham animals (p<0.03)–Fig 4A. All but one of the ten 4.7 hour ischemia animals, which included both occlusion and tourniquet animals, exhibited drop foot for at least 2 days post-operatively. None of the 3.5 hour animals demonstrate drop foot after D1. Tarlov scores were decreased in 3.5 hour occlusion and 4.7 hour tourniquet animals at post-operative time points days 1 through 3 when compared with sham animals (p<0.03)–Fig 4B. This decrease in function was most significant in the 4.7 hour tourniquet animals, and remained depressed throughout the entire survival period, compared to other experimental groups. Interestingly, 4.7 hour occlusion animals demonstrate similar locomotion to 3.5 hour tourniquet animals. The 3.5 hour occlusion animals demonstrate little to no decrease in function/locomotion. Finally, only 25% of the animals in the 4.7 hour ischemia group fully recover by D7; in comparison, 75% of the 3.5 hour ischemia animals fully recover by D7.

### Intraoperative Imaging Directly Observes Post-Occlusive Reactive Hyperemia

Representative 3CCD and infrared (IR) images for baseline, maximum ischemia, 10 minutes post-reperfusion, and 30 minutes post-reperfusion are displayed in Fig 1 (top panels– 3CCD images; middle panels–IR imaging). Both 3CCD and IR mean ROI values are plotted in the bottom two panels (left and right, respectively). 3CCD values (calculated as R-B values) show an almost immediate drop after induction of ischemia, and continue to decrease over the duration of ischemia but at a slow rate. 3CCD values exceed baseline values within the first 10 minutes post-reperfusion and return to almost baseline levels at 30 minutes post-reperfusion. Here, 3CCD imaging (Fig 1 - bottom left) clearly illustrates PORH–an increase in blood flow after release of arterial occlusion either by unclamping of the artery or releasing of the tourniquet. IR imaging values (Fig 1 - bottom right) demonstrate a more progressive and almost linear decrease over the duration of ischemia and do not exhibit the hallmark of PORH after reperfusion, but do return to baseline levels by 30 minutes post-reperfusion.

**Fig 5:**
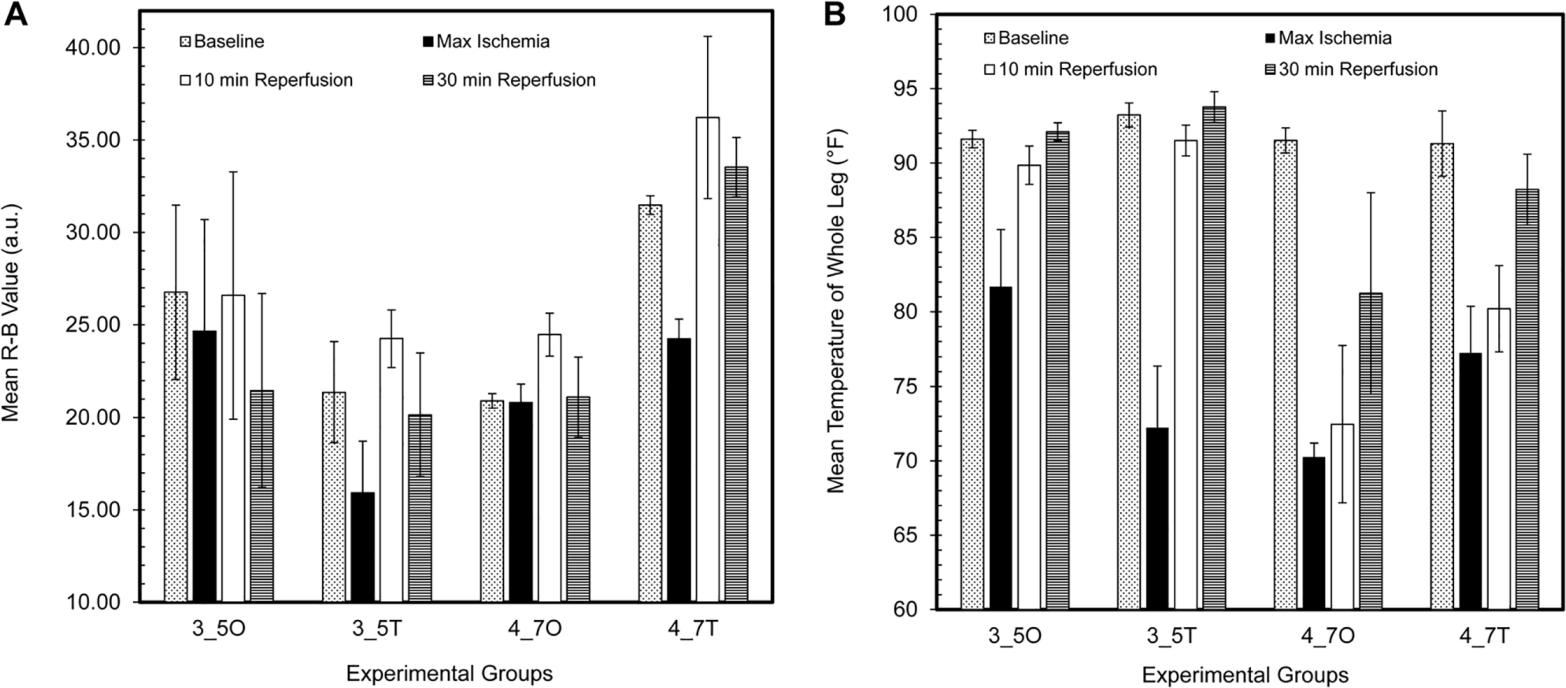
Profiles of operative 3CCD and IR imaging values. Bar plots of mean R-B values derived from 3CCD imaging (A) and mean leg temperatures in °F (B) for all experimental groups at baseline, maximum ischemia, 10 minutes post-reperfusion, and 30 minutes post-reperfusion. Error bars = SEM (standard error of the mean). 3_5O = 3.5 hour occlusion; 3_5T = 3.5 hour tourniquet; 4_7O = 4.7 hour occlusion; 4_7T = 4.7 hour tourniquet.

When comparing the 3CCD imaging profiles of the occlusion and tourniquet experimental groups, there are several notable differences. The degree of severity of the ischemia is evidenced by the decreased R-B values at maximum ischemia for both the 3.5 hour and 4.7 hour tourniquet groups (Fig 5A). Correspondingly, the difference between the R-B vales at maximum ischemia and 10 minutes post-reperfusion is greatest in the tourniquet experimental groups. Here also, we observed PORH, exposed in Fig 5A as an increase in R-B values at 10 minutes reperfusion compared to baseline R-B values.

Though the data was collected on the same animals from the four experimental groups, the IR imaging profiles (Fig 5B) are substantially different from the 3CCD imaging profiles. The degree of severity of the ischemia, exhibited by a decrease in the mean temperature of the leg, is not only noted most significantly in the tourniquet animals but also in the 4.7 hour occlusion animals. Additionally, the magnitude of the difference between the IR values at maximum ischemia and 10 minutes post-reperfusion is not as great as the difference between the IR values at maximum ischemia and 30 minutes post-reperfusion. The mean leg temperature of the 4.7 hour ischemia animals does not return to baseline values and remains significantly less than that of the 3.5 hour ischemia animals (p<0.005). There is also a significant difference between the mean leg temperature at both 5 and 10 minutes post-reperfusion between 3.5 hour ischemia and 4.7 hour ischemia animals (p<0.002).

**Fig 6:**
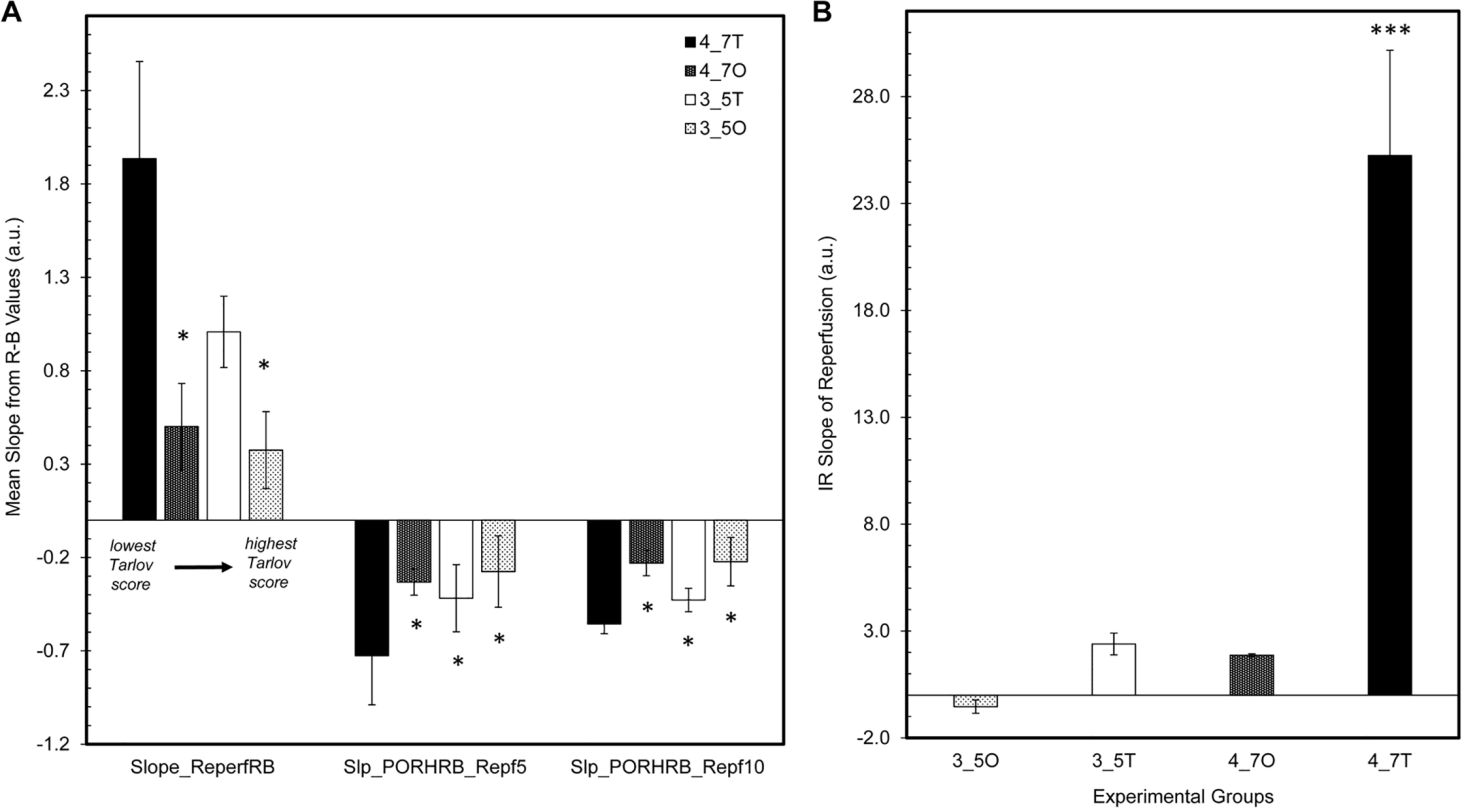
Slopes of reperfusion and post-occlusive reactive hyperemia for 3CCD and IR imaging. Bar plots comparing the IR slopes of reperfusion for all experimental groups (A) and 3CCD slopes of reperfusion, slopes of PORH at 5 minutes reperfusion, and slopes of PORH at 10 minutes reperfusion for all experimental groups (B). Statistically significant differences between 4.7 hour tourniquet animals and other experimental groups are designated by an asterisk (*)–p-value<0.05. Note, for each set of slopes presented in (B), the data is plotted by lowest Tarlov score (i.e. least normal locomotion) and increases to the highest Tarlov score (i.e. most normal locomotion). 3_5O = 3.5 hour occlusion; 3_5T = 3.5 hour tourniquet; 4_7O = 4.7 hour occlusion; 4_7T = 4.7 hour tourniquet.

These changes can also be described by plotting the slopes of reperfusion for both 3CCD and IR imaging (Fig 6A and 6B) and the slopes of PORH at 5 and 10 minutes post-reperfusion for 3CCD imaging (Fig 6A). The 3CCD imaging slope of reperfusion is greatest in tourniquet animals. There is a significant difference between the 3CCD imaging slope reperfusion in the 4.7 hour tourniquet animals and the slope of reperfusion in the 4.7 hour occlusion and 3.5 hour occlusion animals (p<0.05); furthermore, the IR slope of reperfusion in the 4.7 hour tourniquet animals is significantly higher than the IR slopes of reperfusion in all other animal groups (p<0.001). This trend persists for the 3CCD imaging slopes of PORH at both 5 minutes and 10 minutes post-reperfusion (p<0.05).

Based on Pearson’s correlation coefficients, both IR and 3CCD imaging values and slopes are highly correlated with function and mobility and pathology (ρ>0.65 –Table 3). This is less so when comparing IR and 3CCD imaging values and slopes to blood chemistry values (ρ>0.65 –Table 4).

**Table 3:** Correlation of imaging parameters with function and pathology. Pearson's correlation coefficients (ρ) for parameters involving function and pathology. IR = infrared imaging; Rpf = Rpfusion; 3CCD = 3CCD imaging; PORH = post-occlusive reactive hyperemia; D = day.

	Function/Locomotion				Pathology		
Imaging	Tarlov D1	Tarlov D2	Tarlov D7	Drop Foot	Lateral Middle	Medial Middle	Medial Distal
IR Slope Rpf	**-0.672**	**-0.677**	-0.532	0.519	0.364	0.225	**0.728**
IR Whole Leg Rpf10	0.505	0.566	0.391	**-0.775**	0.228	0.400	-0.028
IR Whole Leg Rpf30	0.511	0.532	0.337	**-0.753**	0.088	0.392	-0.094
IR PORH Slope Rpf5	**-0.848**	**-0.848**	-0.525	-0.466	0.526	**0.760**	0.352
IR PORH Slope Rpf10	**-0.764**	-0.398	-0.250	0.220	0.078	0.196	0.386
IR PORH Slope Rpf30	-0.516	**-0.730**	**-0.737**	**0.651**	0.376	0.154	0.363
3CCD Slope Rpf	-0.446	-0.540	-0.609	**0.710**	0.204	0.286	0.574
3CCD Whole Leg Rpf10	0.624	**0.771**	**0.739**	0.342	0.144	0.054	0.077
3CCD Whole Leg Rpf30	0.601	**0.755**	**0.730**	0.272	0.137	0.008	0.031
3CCD PORH Slope Rpf5	**0.652**	**0.889**	0.517	-0.462	-0.158	-0.395	-0.361
3CCD PORH Slope Rpf10	**0.652**	**0.889**	0.517	-0.461	-0.153	-0.395	-0.361
3CCD Whole Leg D1	-0.242	-0.641	-0.633	0.179	**0.776**	0.486	**0.756**
3CCD Whole Leg D3	-0.459	-0.425	-0.384	-0.007	**0.737**	0.603	**0.712**

**Table 4:** Correlation of imaging parameters with blood chemistries. Pearson's correlation coefficients (ρ) for parameters involving blood chemistries. IR = infrared imaging; Rpf = Rpfusion; 3CCD = 3CCD imaging; PORH = post-occlusive reactive hyperemia; D = day.

	Blood Chemistry									
Imaging	BUN Max Isc	ALT Max Isc	Creatine Max Isc	LDH Max Isc	CK Max Isc	BE Max Isc	BUN Rpf	Creatine Rpf	CK Rpf	BE Rpf
IR Slope Rpf	0.441	-0.448	-0.399	-0.084	-0.105	0.399	0.480	-0.359	0.411	0.619
IR Whole Leg Rpf10	**-0.663**	0.343	-0.418	-0.186	0.241	-0.460	-0.614	-0.454	0.018	**-0.761**
IR Whole Leg Rpf30	**-0.666**	0.432	-0.361	-0.164	0.219	-0.590	-0.604	-0.371	-0.072	**-0.890**
IR PORH Slope Rpf5	-0.374	0.347	-0.567	0.095	0.114	-0.188	-0.315	-0.527	0.320	-0.513
IR PORH Slope Rpf10	0.501	0.343	-0.401	0.082	-0.166	0.216	0.532	-0.344	0.144	0.251
IR PORH Slope Rpf30	0.534	0.432	-0.452	-0.015	-0.390	0.326	0.571	-0.399	0.072	0.379
3CCD Slope Rpf	0.034	-0.141	-0.404	0.046	-0.178	0.318	0.112	-0.281	0.207	0.473
3CCD Whole Leg Rpf10	0.635	0.444	0.166	0.508	-0.107	0.343	**0.660**	0.160	0.351	**0.824**
3CCD Whole Leg Rpf30	0.589	0.408	0.236	0.484	-0.067	0.411	0.622	0.230	0.382	**0.803**
3CCD PORH Slope Rpf5	-0.648	-0.026	-0.034	-0.149	0.381	-0.324	-0.644	-0.048	-0.025	-0.349
3CCD PORH Slope Rpf10	-0.642	-0.040	-0.045	-0.141	0.367	-0.319	-0.635	-0.059	-0.038	-0.355
3CCD Whole Leg D1	0.263	-0.541	**-0.790**	**-0.741**	**0.658**	0.067	0.392	**-0.740**	**0.838**	0.264
3CCD Whole Leg D3	0.629	**-0.755**	**-0.839**	**-0.663**	**0.722**	-0.053	**0.756**	**-0.830**	**0.812**	0.200

### Predicting Full Recovery of Locomotion Post-operatively after I/R Injury

**Fig 7:**
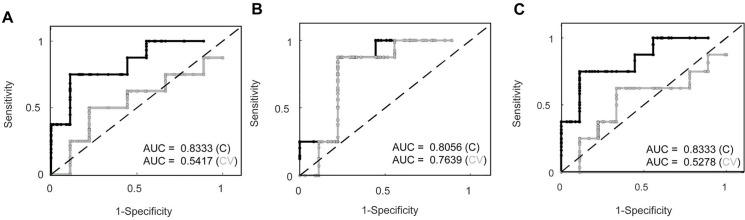
PLSDA models to predict return to normal locomotion for all data, imaging only data, and non-imaging data sets. Receiver operating curves for both calibration and cross-validation data sets. Area under the curve (AUC), a surrogate for accuracy, is displayed for each curve. A) PLSDA model generated from all parameters, but excluding outcome parameters. B) PLSDA model calculated from only imaging parameters. C) PLSDA model generated from all non-imaging parameters, but excluding outcome parameters. The calibration data is plotted in black and the cross-validation data is plotted in gray.

Finally, as demonstrated in Fig 7A–7C, we were able to predict whether or not the affected limb would fully recover by post-operative D7. In a PLSDA model based on all parameters but excluding outcome parameters (Fig 7A), the training model performed decently (62.5% sensitivity and 88.9% specificity) but the same was not true for the cross-validated model (62.5% sensitivity and 55.6% specificity). Next, we calculated a PLSDA model based on only imaging parameters (Fig 7B); the training model and the cross-validated model both performed very well with 87.5% sensitivity and 77.8% specificity for the training model and 87.5% sensitivity and 66.7% specificity for the cross-validated model. Lastly, we generated a PLSDA model using all non-imaging parameters but excluding outcome parameters (Fig 7C). The training model performed comparably to the training model with all parameters (62.5% sensitivity and 88.9% specificity), but the cross-validation model did not perform as well as the cross-validation model using imaging parameters only (62.5% sensitivity and 66.7% specificity). An area under the curve (AUC) of greater than 0.8 was observed in all training models; only the cross-validation model made from imaging parameters only approached an AUC of 0.8.

## Discussion

Parameters derived from 3CCD and IR imaging show promise as surrogate outcome measures for tissue oxygenation and perfusion, as it relates to ischemia/reperfusion injury and return to normal locomotion. In addition, we confirmed that 4.7 hours is the upper limit for tolerated critical ischemia before disability is permanent in swine. Translating this information into clinical scenarios will be an important next step to determine the upper limit of ischemia in humans as it relates to function. Likewise, using these imaging technologies to identify the extent of ischemia and reperfusion in a clinical setting will have important implications.

The hind limb ischemia model utilized in this study validates our development of a critical acute limb ischemia model. Interestingly, the 4.7 hour tourniquet animals had more profound and prolonged disability compared to the 4.7 occlusion animals despite complete proximal vessel occlusion; both experimental arms demonstrated a meager 25% recovery to full locomotion post-operatively. The difference in disability between the 4.7 hour tourniquet group and the 4.7 hour occlusion group could be attributable to direct vascular injury from the vascular clamp, but most likely is the result of reduced or nonexistent collateral flow below the tourniquet compared to direct vessel occlusion. This may also be due in part to direct tissue injury from the tourniquet itself (focal crush injury where tourniquet was applied). This additional, direct injury in the tourniquet animals is particularly evidenced by the slope of reperfusion as determined by 3CCD imaging. We postulate that the 3CCD imaging slope of reperfusion is greatest in tourniquet animals because vessel occlusion of all vessels in the limb is more complete in the tourniquet animals compared to the occlusion animals; that is, some collateral flow was maintained below vessel occlusion compared to the more distally placed tourniquet. As such, vessel recovery and reperfusion was more prolonged in the tourniquet groups as compared to the occlusion groups.

Recovery from tissue ischemia is largely dependent on tissue microcirculation. Though microcirculation consists of the smallest blood vessels, including arterioles, capillaries, and venules, it is responsible for tissue oxygenation and ultimately tissue (and organ) health.[14] During critical ischemia, microcirculatory endothelial cells fail to perform their regulatory function, smooth muscle cells lining arterioles lose their ability to regulate perfusion, and red blood cells aggregate. The nitric oxide system is greatly impaired resulting in reduced vasodilation, diminished revascularization, and inhibition of critical cell signaling; a reduction of nitric oxide activity has been implicated in delayed wound healing.[15, 16] Additionally, ischemia activated leukocytes generate reactive oxygen species that barrier function in the microcirculation. It is the agglomerate of these impairments to the microvasculature that contribute to acute ischemia and in more severe cases, organ failure and death. In a study by Sakr et al., a lower percentage of perfused microcirculation was associated with poorer outcome, i.e. multiple organ failure and death.[17]

Post-occlusive reactive hyperemia (PORH) can be used to detect overall changes in microcirculation by measuring the response of distal microcirculation after a period of proximal occlusion, where the applied pressure of the cuff exceeds the systolic pressure of the patient. The extent to which the blood flow increases following cuff release can be used to quantify several microcirculation parameters, including the peak raw value, peak minus baseline, percentage increase from baseline, area under the curve, and time to peak hyperemia.[18] PORH monitoring has been used to evaluate microcirculation in arterial occlusive disease [19–21], peripheral vascular disease [22–24], and spinal cord injury [25–27]. PORH measurements are often made on the forearm.[18, 28] Previous studies of PORH have used laser Doppler imaging, laser speckle imaging, ultrasonic imaging, photoacoustic microscopy, and near-infrared imaging to monitor changes in microcirculation non-invasively.[29–33] Near-infrared imaging, also known as indocyanine green angiography, requires the injection of a dye, such as indocyanine green, and the use of specialized near-infrared cameras to capture the extent of vascularity within flaps during reconstructive procedures.[34, 35] Unfortunately, dye might not be readily available in austere environments, injection may cause anaphylactic reactions[36]; furthermore, indocyanine green angiography includes high cost, increased duration of surgical procedure, and inferiority of performance when compared to clinical assessment.[37] Most importantly, while indocyanine green-based imaging modalities may discern whether or not tissue is being perfused, there is no avenue for determining actual tissue oxygenation which may result in false conclusions regarding the viability of certain tissues.[38] Laser Doppler imaging (LDI), founded on the idea that changes in blood flow can be measured via backscattering of a laser beam when it interacts with moving red blood cells, has been utilized extensively over the past 30 years to visualize tissue perfusion; while the non-invasive nature of LDI is highly desirable, it suffers from lack of standardization.[29] Laser speckle contrast imaging (LSCI) presents an index of blood flow not unlike LDI, but is able to provide near real-time feedback while LDI scanning suffers from a reduction in temporal resolution[39]; however, LSCI only penetrates approximately 300 microns into tissue while LDI will provide details about tissue approximately 1 cm below the surface.[29, 39] Further investigation of other imaging modalities to ascertain tissue viability is warranted.

In this study, using 3CCD and IR technology, our data shows that we can successfully monitor PORH in acute and critical limb ischemia. This is possible because 3CCD imaging and IR imaging are measuring tissue oxygenation and perfusion simultaneously. Briefly, for 3CCD imaging, a color image is reconstructed and recorded using red, green, and blue bandpass filters in front of three separate monochrome CCDs. The individual colors can be combined, subtracted and otherwise manipulated to enhance the contrast of an image so that detection is sensitive to molecules of interest. 3CCD imaging enhancement arises directly from the absorption properties of oxygenated and deoxygenated hemoglobin (HbO_2_ and Hb, respectively).[10] We have proven experimentally that 3CCD imaging is a viable technology to monitor intraoperative renal parenchymal oxygenation [12] and bowel ischemia.[40, 41] With IR imaging, assessment of tissue perfusion can be determined based on the principles of thermography.[42] These differences can be quantified using a digital IR camera in a non-invasive manner. We have previously studied the application of noninvasive digital IR imaging in models of cerebral and renal perfusion.[43, 44]

The comparison of 3CCD imaging and IR imaging in this study demonstrates our ability to monitor not only dysfunction of the microcirculation in a model of acute ischemia but in entire limb as well. In this study, the slope of reperfusion (a variation of time to peak hyperemia) for both 3CCD imaging and IR imaging could be utilized as a surrogate measure for severity of acute limb ischemia. There is currently no PORH parameter that closely resembles what we term the slope of PORH, i.e. the difference between the 3CCD and IR imaging maximum reperfusion values and their corresponding 5 or 10 minute post-reperfusion values divided by the duration of the post-reperfusion period; however, the slope of PORH values for both 3CCD and IR imaging are most correlated to locomotion within the first 48 hours post-operatively as shown in Table 3. Importantly, to our knowledge, this is the first demonstration of whole limb monitoring for tissue perfusion and oxygenation measurements simultaneously.

Perhaps the PLSDA model developed with imaging parameters in this study most eloquently demonstrates the potential of non-invasive imaging, specifically 3CCD and IR imaging, to predict full recovery of locomotion after a potential critical ischemic injury. This has important clinical implications as these imaging capabilities will allow clinicians to determine the extent of reperfusion injury and tissue necrosis, which can help guide surgical debridement. In addition, 3CCD and IR imaging may have a role in determining if a limb requires amputation should there be no functional recovery. Finally, these imaging techniques can assist clinicians in discussions with patients regarding functional outcome after IRI. We have also confirmed the development of a large animal hind limb model for critical ischemia using both a tourniquet and direct vessel occlusion technique. The results presented herein are preliminary and would need to be further demonstrated on a larger scale with more animals over a longer period of recovery and validated with a clinical trial. This model will serve as the basis for further studies on ischemia-reperfusion injury and ways to mitigate its effects using 3CCD and IR imaging to guide treatment.

In summary, as suspected, 4.7 hour tourniquet animals demonstrated the most significant decrease in limb recovery, as evaluated by function/mobility and muscle histopathology. This supports the previously postulated critical ischemia of approximately 4.7 hours. In animals that were subjected to a shorter duration of ischemia, the injury was not severe enough to impair function/locomotion for the entire survival period. Furthermore, non-invasive 3CCD and IR imaging technologies accurately monitor whole limb perfusion and oxygenation, enabling visualization of PORH; this in turn enables accurate prediction of full function/locomotion recovery post-operatively. While IR imaging correlates with immediate outcome (≤ 24 hours post-operative), 3CCD imaging correlates with longer term outcome (≥ 48 hours post-operative). This indicates the need for the complementary use of both imaging technologies, which outperform standard clinical parameters when identifying highest risk for debilitating ischemia/reperfusion injury as it relates to tissue perfusion and oxygenation. Finally, 3CCD imaging and IR imaging offer the added advantage of easy incorporation into small hand-held devices, which can be deployed in a variety of clinical settings including the battlefield, medical transport, and modular medical treatment facilities.

## Supporting Information

S1 File Minimal data set. (TXT)
